# Nurses’ burden caused by sleep disturbances of nursing home residents with dementia: multicenter cross-sectional study

**DOI:** 10.1186/s12912-020-00478-y

**Published:** 2020-09-09

**Authors:** Denise Wilfling, Martin N. Dichter, Diana Trutschel, Sascha Köpke

**Affiliations:** 1 Institute of Social Medicine and Epidemiology, Nursing Research Group, University of Lübeck, Ratzeburger Allee 160, 23538 Lübeck, Germany; 2 Institute of Nursing Science, University Hospital Cologne, Gleueler Straße 176-178, 50935 Cologne, Germany; 3 German Center of Neurodegenerative Diseases, Stockumer Straße 12, 58453 Witten, Germany; 4 School of Nursing Science, Witten/Herdecke University, Alfred-Herrhausen-Straße 50, 58455 Witten, Germany

**Keywords:** Nurses’ burden, Dementia, Sleep wake disorders, Nursing home, Survey, Germany

## Abstract

**Background:**

Sleep disturbances are common in people with dementia. In nursing homes, this is frequently associated with residents’ challenging behavior and potentially with nurses’ burden. This study examined nurses’ burden associated with nursing home residents’ sleep disturbances.

**Methods:**

A multicenter cross-sectional study was conducted. Nurses’ burden associated with residents’ sleep disturbances was assessed using the Sleep Disorder Inventory (SDI). Additionally, the proportion of nurses’ total burden associated with sleep disturbances of residents with dementia was assessed. A linear mixed regression model was used to investigate the association with nurses’, residents’ and institutional characteristics.

**Results:**

One hundred eleven nurses from 38 nursing homes were included. 78.4% stated to be regularly confronted with residents’ sleep disturbances during nightshifts, causing distress. The mean proportion of nurses‘ total burden caused by residents‘ sleep disturbances was 23.1 % (SD 18.1). None of the investigated characteristics were significantly associated with nurses’ total burden.

**Conclusions:**

Nurses report burden associated with sleep disturbances as common problem. There is a need to develop effective interventions for sleep problems and to train nurses how to deal with residents’ sleep disturbances.

## Background

Dementia is a disease associated with several cognitive, neuropsychiatric and functional symptoms, affecting all areas of daily living and functioning [1]. Worldwide, more than 24 million people are affected and numbers are increasing as people are getting older. In western countries, a prevalence of 6% among people of 60 years or older has been estimated [2]. There are currently around 1.5 million people with dementia in Germany with more than 300,000 new cases each year [3]. The prevalence of people with major cognitive impairment in German nursing homes was found to be 60% in a recent multi-center study [4].

Caring for people with dementia is a challenging task. Behavioral and psychological symptoms of dementia (BPSD) are common and include a variety of symptoms like aggressive behavior [5], failing verbal communication [6], wandering around [7] and apathic behavior [8]. In Germany, a study on the prevalence of BPSD in nursing home residents with dementia have found a prevalence of 60% [9].

BPSD are associated with distress and challenges, not only for the affected persons, but also for nurses. Nurses regularly confronted and challenged with BPSD tend to be more burdened and less balanced [10]. BPSD has been identified as a factor that affects nurses’ satisfaction, general health, and ability to work [11–13]. Caring for residents with BPSD also indirectly increases costs, because they require more caregiving hours than residents without BPSD [14].

Sleep disturbances are common in people with dementia including increased number and duration of awakenings, increased percentage of time spent in stage one sleep, and increased day time sleep [15]. In different settings, prevalences of sleep disturbances of up to 40% have been found in people with dementia [16–18]. A recent prevalence study conducted in the same 38 nursing homes recruited for this study, showed a mean of 23% of residents with sleep disturbances. Marked differences between centers were found with prevalences ranging from 0 to 85% [19].

Although sleep problems in older people are very common, they differ from sleep problems in people with dementia. Because of degenerative changes in dementia, a reversal of sleep-wake cycle and increased daytime sleep is common in people with dementia [20, 21], further challenging care routines and burdening carers [22, 23] and worsening cognitive decline and increasing BPSD [20, 21].

Therefore, there is a clear need to determine the extent of emotional distress of nurses associated with sleep disturbances in people with dementia. Considering the lack of high quality data especially from Germany, the aim of this study is to examine nurses’ burden associated with sleep disturbances of people with dementia in German nursing homes and possible associated factors.

## Methods

### Study design

A multicenter cross-sectional study was conducted within a randomly selected group of nursing homes from Northern Germany. The methods have been described in detail in a previous publication, presenting the prevalence of sleep disturbances in 1187 residents with cognitive impairment [19].

### Sample

As the study is exploratory, there was no valid reference point for sample size calculation. We followed the recommendation by Schneider et al. 2010 [24], which states that the number of observations should be at least 20 times greater than the number of variables included in linear regression analyses. We were aiming for a sample of minimum 100 nurses to be reasonably sure to achieve precise estimates for variables.

### Recruitment

The recruitment procedure has been described before [19]. Nursing homes from Northern Germany were randomly selected from nursing home registers between June and December 2017. As a first step, in nursing homes interested in participation, nursing home managers were contacted with verbal and/or written information and asked for informed consent before participating in the study. In a second step, nurses were identified by the head nurse and approached by the study team. Only nurses who were regularly working in night shifts were included. Nurses had to have worked at least three nightshifts within the last 3 months prior to data collection. All participating nurses gave informed consent.

### Procedures

Procedures have been reported in detail before [19]. All data concerning nursing homes, residents, and nurses were assessed using pseudonymization on the level of nursing homes.

### Measurements

Three standardized questionnaires were applied: (1) questionnaire for nursing homes, (2) questionnaire for nurses (3) questionnaire about residents (proxy rating by nurses) [19]. Nursing home and nurses’ characteristics were assessed using two questionnaires, developed and piloted by the research group. The nursing home questionnaire was completed by the nursing home manager and comprised questions on ownership of the nursing home (non-profit, private, public, clerical), number of wards and residents per nursing home (continuous), the care concept of the nursing home (no specific concept, dementia-specific care, others) and presence of a institutional guideline to deal with sleep disorders (yes/no).

The questionnaire for nurses (see appendix) included demographic and professional characteristics, exposure to residents’ sleep disturbances, burden associated with sleep disturbances, knowledge about interventions to avoid sleep disturbances (yes, no) and source of this special knowledge (nursing training, academic nursing training, continuous nursing education, personal work experience, self-study).

For the assessment of nurses’ burden, the relevant subscale of the Sleep Disorders Inventory (SDI) [25] was applied. Nurses were asked: “How emotionally distressing is this for you?” for each of the night time behaviors of a particular resident. Response options were “not at all”, “minimally”, “mildly”, “moderately”, “severely” and “extremely”. Additionally, the proportion of nurses’ total burden associated with residents’ sleep disturbances was assessed. Here, nurses could enter a value between 0 and 100 (%).

### Data analysis

Due to pseudonomization on nursing home level, questionnaires could not be assigned to individual nurses or residents. As nurses’ and residents’ data could be assigned to specific nursing homes, “care dependency level” and “SDI score” were included on the level of residents. These variables were merged with nurses’ data using the average value for nursing homes corresponding to nursing homes.

Sample characteristics and nurses’ burden (ordinal data of the SDI) were computed using descriptive statistics. For the investigation of possible associated factors of nurses’ burden, mixed regression models were performed for random structured data. Having three data sets corresponding to the measurements of (1) nursing homes, (2) nurses, and (3) nursing home residents, merging of all datasets was necessary to address the scientific question. For analysis, only complete data sets were considered, and data sets were excluded based on missing values.

The primary outcome of interest was nurses’ burden related to residents’ sleep disturbances (0–100%). Hence, the dependent variable of the generalized linear model was mean percentage of nurses’ total burden.

The random effects institution was included to adjust for correlations of residents, nurses and the nursing home. To investigate associations of assumed predictor variables, the following data were included as fixed effects:

- Nurses’ characteristics: special knowledge about interventions to avoid sleep disturbances (yes/no),working hours per week (continuous)
- Institutional characteristics: existence of an institutional guideline on sleep disturbances (yes/no), special dementia care concept (yes/no)
- Resident characteristics: resident’s mean care dependency level within a nursing home (1–5), mean SDI score of all residents within a nursing home (0–85)

Statistical analyses were performed using the software SPSS v. 22 [26] (descriptive statistics) and R Version 3.2.4 [27] (generalized mixed regression model).

## Results

Thirty-eight nursing homes and 1187 residents with cognitive impairment were included. Numbers of residents with cognitive impairment differed between nursing homes (mean 31 ± 28, Range 4–146). Sleep disturbances were identified for 23% of residents with pronounced differences between centers, ranging from 0 to 85%. Detailed characteristics of nursing homes and residents have been reported before [19].

The majority of nursing homes were working with fixed night staff. Of these all 196 nurses were asked to participate in the trial and finally 111 nurses consented and took part in the survey (mean number per nursing home: 2.8 ± 2.4, Range 0–9). Characteristics of nurses are shown in Table 1. Eighty-seven (78.4%) stated to be regularly confronted with residents’ sleep disturbances during night shifts, causing burden. The proportion of nurses‘total burden related to resident‘s sleep disturbances was on average 23.1% (± 18.1), with pronounced differences ranging from 0 to 70%. Having special knowledge about interventions to avoid sleep disturbances was reported by 70.3% of nurses.

**Table 1:** Characteristics of nurses

Nurses	***N*** = 111
Age, number (%)	
20–30 years	14 (13)
31–40 years	31 (28)
41–50 years	32 (29)
> 50 years	32 (29)
Women, number (%)	90 (81)
Contract hours, number (%)	
Full time	49 (44.1)
Part time	62 (55.9)
Years working in elderly care, mean (SD)	18.6 (±9.3)
Level of healthcare training, number (%)	
Nurse registered	77 (69.4)
Nurse assistant	34 (30.6)
Special dementia or geriatric nursing training number (%)	1 (1.1)
Confrontation with sleep disturbances, number (%)	87 (78.4)
Proportion of nurse’s total burden caused by sleep disturbances, mean (SD)	23.1 (±18.1)
Special knowledge about interventions to avoid sleep disturbances, number (%)	78 (70.3)
Knowledge sources, number (%)	
Nursing training	40 (36)
Academic Nursing Training	1 (0.9)
Continuous Nursing education	45 (40.5)
Personal work experience	63 (56.8)
Self study	21 (18.9)

Data are the mean (SD) or number (%)

### Associated factors of nurse’s burden caused by sleep disturbances

Having three data sets corresponding to the measurements of (1) 38 nursing homes, (2) 111 nurses, and (3) 1187 residents in nursing homes, merging of all datasets was necessary to address the scientific question. Two nurses were excluded based on missing values in the variables included in the model. Therefore, the final dataset consisted of 109 nurses for analysis.

Table 2 shows the results of the linear mixed model on the percentage of nurses’ total burden to identify associated factors. None of the chosen variables had a significant association with nurses’ burden, neither special dementia care concept (*p* = 0.27), average SDI score of all residents within a nursing home (*p* = 0.39), or existence of an institutional guideline on sleep disturbances (*p* = 0.42).

**Table 2:** Possible associated factors with percentage of nurses’ total burden caused sleep disturbances

Overall ***n*** = 109^**a**^ nurses			
	Estimates	CI 95%	***p***-values
Special knowledge about interventions to avoid sleep disturbances	−2.12	−10.19 – 5.95	0.61
Working hours per week	−2.32	−9.37 – 4.73	0.52
Existence of an institutional guideline on sleep disturbances	−5.35	−18.17 – 7.48	0.42
Special dementia care concept	5.83	−4.27 – 15.93	0.27
Average resident’s care dependency level	0.74	−11.59 – 13.08	0.91
Average SDI score of all residents within a nursing home	0.33	−0.41 – 1.07	0.39

^a^Final data set for the regression model after excluding missing values

Estimates = Estimates of the linear mixed regression model

CI 95% = Confidence interval 95%

*p*-values = computed *p* value of t-test for regression coefficient, level of significance = 5%

## Discussion

The aim of the present study was to determine nurses’ burden associated with sleep disturbances of nursing home residents with cognitive disorder. The included nursing homes in the present study differed in size, specific living facilities and care concept for people with cognitive disorders. We found that nurses’ burden was common with 78.4% stating to be regularly confronted with resident’s sleep disturbances during night shifts and 80.1% stated to be emotional distressed mildly to severely. Thus, sleep disturbances clearly represent a very common problem for nurses, causing burden and distress. We found a mean proportion of nurses’ total burden associated with residents‘sleep disturbances of 23.1%. Balzer et al. 2013 [28] conducted a survey including 160 nurses from different wards in a hospital setting, assessing challenges and burden of caring for patients with dementia and found similar results. The mean proportion of nurse’s total burden caused by caring for people with dementia was 37.5% (Interquartile Range 20–60) and nurses stated that BPSD of patients with dementia was one of the main reasons for burden.

Schmidt et al. 2012 [11] conducted a cross-sectional study including 731 registered nurses and nursing aides in 56 German nursing homes to assess nurses’ BPSD-related distress. Data was assessed using a BPSD-related distress index and showed that nurses experienced a moderate level of distress. Although this study did not focus on sleep disturbances of residents with dementia, the reported burden is comparable to our study.

We assessed sleep disturbances using the SDI and found a prevalence of 23% overall, the most common sleep disturbances being getting up during night, wandering around and activities at night. We only included residents with cognitive impairment in our study. As already shown in Schäufele et al. 2009 [29] and Hardenacke et al. 2011 [30], BPSD is present in 90% of residents with dementia, but also in 30% of patients without dementia. Accordingly, it can be assumed that also residents without dementia are affected by sleep disturbances, which further increases challenges in the care process and nurses’ burden.

Although guidelines propose the use of non-pharmacological interventions for BPSD including sleep problems [31, 32], residents are frequently treated with antipsychotics and physical restraints. Consequences of this treatment can be increased mortality, accidental falls and social isolation [33].

Effective treatment of BPSD as well as sleep disturbances depends on whether causes and determinants of symptoms and problems are identified, so that treatment can target these causes [34]. Determinants can be divers, e.g. neurodegeneration, type of dementia, severity of cognitive impairments, declining functional abilities and also, nurses’ distress, communication and tailored activities. Detection and understanding of these factors are necessary for the effectiveness of person-centered interventions to avoid or reduce BPSD, with nurses playing a key role. To assess BPSD including sleep disturbances and to act preventive, expert knowledge and advanced skills are necessary [35].

In our study, 78% of nurses stated to have knowledge about interventions to avoid sleep disturbances. The most common source of knowledge was reported to be personal working experience, followed by continuous nursing education and nursing training. These results are slightly disturbing as these sources in a rapidly evolving field, are surely not the most adequate source of knowledge compared to recent research findings as e.g. from practice guidelines. Although most nurses claim to know about interventions to manage sleep disturbances, the emotional burden caused by sleep disturbances cannot be ignored. A quarter of the total burden of caregiving was reported to be caused by sleep disturbances. This raises the question whether there is a lack of knowledge on effective interventions, specifically non-pharmacological interventions to avoid sleep disturbances and an increased need for training.

Different from the present study, Balzer et al.2013 [28] identified hospital nurses’ uncertainties in the care of people with dementia with less than 70% of nurses stating to feel safe and confident in dealing with BPSD and reporting a need for more training. The reason for these differences might be the different setting and the higher prevalence of people with dementia and therefore more experiences in dementia care and care of people with BPSD in the present study.

None of the investigated variables demonstrated a significant association with nurses’ burden. Schmidt et al. 2012 [11] found the same results by assessing the association between socio-demographic and occupational characteristics on BPSD-related distress. Also, in these study personal factors like years working in nursing, working hours or the qualification as nursing aid or registered nurse did not show significant associations with nurses’ burden. Only nurses’ age was found as significantly associated with burden.

Our results concerning possible associated factors with burden caused by sleep disturbances raises the questions why we were not able to identify associated factors and which factors are associated. One factor might be nurses’ attitudes to dementia and sleep disturbances in general which we have not assessed as possible factor. A previous study [36] found that people with dementia are often perceived more negative by nurses. The consideration of nurses’ attitudes seems to be important, because individual attitudes are a crucial associated factor.

Furthermore, it must be considered that existing associations between resident and institutional characteristics may have been overlooked due to the relatively small sample size. Therefore, results of this exploratory study should be verified in further studies. In summary, the present study documented the presence of nurses’ burden associated with sleep disturbances of residents with dementia. As there is a lack of studies assessing nurses’ burden caused by sleep disturbances of nursing home residents with dementia, our study provides important data to improve the well-being of both nurses and people with dementia and offers the basis for the development of future interventions.

### Limitations

Our methodological approach was accompanied by various limitations. First, our study is based on an exploratory design, that is, it aims to generate hypotheses but not to test them. Estimates in the generalized mixed regression model have wide confidence intervals limiting the relevance of the results, but it has to be taken into account that this is an exploratory study, and therefore we are only able to generate hypotheses. Therefore, the identified associations must be interpreted with caution and verified in further studies Second, we missed the assessment of possibly relevant factors like nurses’ attitudes. Future studies should therefore consider factors such as nursing attitudes. Third, a range of one to nine nurses within a nursing home were included. This unbalanced data structure of nurses within nursing homes is a further limitation. Fourth, a limitation is the assessment of cognitive disorders which was not conducted by using standardized instruments in this study, rather based on documentation in medical records.

## Conclusions

The prevalence of sleep disturbances in people with dementia in Germany is comparable to reports from other countries. Little is known about how nurses judge and apply interventions, about interventions’ circumstances and challenges within care processes. BPSD leads to a decreased quality of general health, lower workability, increased burnout levels [11], depression, anxiety, and sleep disturbances in nurses [37]. In consequence this can result in sick leave, turnover and higher economic costs [1, 38].

Although most nurses claim to know about interventions to manage sleep disturbances, the emotional burden caused by sleep disturbances is a common problem. Therefore, there is a need to develop effective interventions based on recommendations included in the MRC framework [39] and to train nurses on how to implement this in daily care routine to avoid sleep disturbances in residents with dementia and finally reduce nurses’ burden and distress. Interventions aimed at preventing and identifying residents with sleep disturbances may ease the burden of caregiving. Moreover, the investigation of possible associated factors with burden caused by sleep disturbances must be continued in further studies. Because of the explorative character of this study, results should be verified in further studies.

## Supplementary information

**Additional file 1.**

## Data Availability

All data generated or analysed during this study are included in this published article.
