# Supplementary material for: Nurses’ burden caused by sleep disturbances of nursing home residents with dementia: multicenter cross-sectional study
Source: BMC Nurs. 2020 Sep 9;19:83. doi: 10.1186/s12912-020-00478-y (PMC7487724; doi:10.1186/s12912-020-00478-y)
Supplement: Supplementary file 1 — Additional file 1. [file 12912_2020_478_MOESM1_ESM.docx]

**Nurses’ burden caused by sleep disturbances of nursing home residents with dementia**

Date: _____________

**Part 1: Characteristics**

1. **Gender**

🞏 male 🞏 female

1. **How old are you?**

🞏 <20 years 🞏 20-30 years 🞏 31-40 years 🞏 41-50 years 🞏 >50 years

1. **Do you work full or part time?**

🞏 full time

🞏 part time: ______ hours/week

1. **How many years have you been working in elderly care?**

______ years

1. **What is your level of healthcare training?**

🞏 elderly care nurse

🞏 elderly care assistant

🞏 registered nurse

🞏 nurse assistant

🞏 trainee

🞏 Other: _________________________________________

1. **Do you have any special qualifications in dementia or geriatric nursing?**

🞏 yes: ____________________________________________________

🞏 no

**Part 2: Sleep disturbances in people with dementia**

1. **Are you confronted with sleep disturbances in people with dementia during daily care?**

🞏 yes

🞏 no

1. **How emotionally distressing do you find this behavior?**

🞏 not at all

🞏 minimally (mostly no influence on the workflow)

🞏 mildly (mostly no influence on the workflow, little additional time required)

🞏 moderately (disrupts the work routine, takes time)

🞏 severely (disruptive, negative influence on staff and other residents)

🞏 very severely/extremely (very annoying, main cause of stress for staff and other residents, takes up time that would actually be intended for other residents or activities)

1. **Please estimate the proportion of your total work burden caused by sleep disturbances in people with dementia?**

______ %

1. **Do you have special knowledge about interventions to avoid sleep disturbances in people with dementia?**

🞏 yes

🞏 no

(if „yes“ go to question 11, otherwise go to question 12)

1. **Where did you get this knowledge from?**

🞏 nursing training

🞏 academic nursing training

🞏 continuous nursing education,

🞏 personal work experience

🞏 self study

🞏 other: ___________________________________________________

1. **Please briefly state the interventions to avoid sleep disturbances in people with dementia you are aware of?**

**Thank you for your cooperation!**
